# Anisotropic light scattering from myelinated axons in the spinal cord

**DOI:** 10.1117/1.NPh.7.1.015011

**Published:** 2020-03-10

**Authors:** Damon DePaoli, Alicja Gasecka, Mohamed Bahdine, Jean M. Deschenes, Laurent Goetz, Jimena Perez-Sanchez, Robert P. Bonin, Yves De Koninck, Martin Parent, Daniel C. Côté

**Affiliations:** a CERVO Brain Research Center, Québec City, Québec, Canada; b Center for Optics, Photonics and Lasers, Québec City, Québec, Canada; c University of Toronto, Leslie Dan Faculty of Pharmacy, Toronto, Ontario, Canada

**Keywords:** optogenetics, tissue optics, Monte Carlo, spinal cord, optical properties, myelin

## Abstract

Optogenetics has become an integral tool for studying and dissecting the neural circuitries of the brain using optical control. Recently, it has also begun to be used in the investigation of the spinal cord and peripheral nervous system. However, information on these regions’ optical properties is sparse. Moreover, there is a lack of data on the dependence of light propagation with respect to neural tissue organization and orientation. This information is important for effective simulations and optogenetic planning, particularly in the spinal cord where the myelinated axons are highly organized. To this end, we report experimental measurements for the scattering coefficient, validated with three different methods in both the longitudinal and radial directions of multiple mammalian spinal cords. In our analysis, we find that there is indeed a directional dependence of photon propagation when interacting with organized myelinated axons. Specifically, light propagating perpendicular to myelinated axons in the white matter of the spinal cord produced a measured reduced scattering coefficient ($\mu_{s}^{\prime }$) of $3.52\pm 0.1\;{\mathrm{mm}}^{-1}$, and light that was propagated along the myelinated axons in the white matter produced a measured $\mu_{s}^{\prime }$ of $1.57\pm 0.03\;{\mathrm{mm}}^{-1}$, across the various species considered. This 50% decrease in scattering power along the myelinated axons is observed with three different measurement strategies (integrating spheres, observed transmittance, and punch-through method). Furthermore, this directional dependence in scattering power and overall light attenuation did not occur in the gray matter regions where the myelin organization is nearly random. The acquired information will be integral in preparing future light-transport simulations and in overall optogenetic planning in both the spinal cord and the brain.

## 1 Introduction

Optogenetics, the use of light and genetic engineering to probe and manipulate cell activity, is an important emerging technology that is instrumental in decoding the functional organization of brain tissue. More recently, the technique has also shown promise in the study of the spinal cord and peripheral nervous system.^1^^–^^3^ In the context of motor guidance, optogenetic tools have been used to establish the role played by individual neural populations in motor navigation and have shown potential for restoring function after spinal cord injury or motor neuron disease.^4^^–^^7^ In the study of sensory and pain processing, experiments involving targeted optical stimulation have greatly expanded our knowledge about the connectivity and function of peripheral and spinal sensory neurons.^8^^–^^12^ *In vivo* control of somatosensory circuits continues to enable researchers to study behavioral consequences of stimulation in specific classes of neurons under both normal and pathological conditions without confounding effects of genetic ablation or pharmacological intervention.^13^^,^^14^

Nonetheless, a question that will continue to affect interpretation of optogenetic experiments is whether a lack of physiological response to the light stimuli comes from abnormalities in neural circuit activity or failure of the stimulation itself. Optogenetic control of nerve cell activity relies on the expression of light-sensitive proteins called opsins, which generate depolarizing or hyperpolarizing currents when exposed to light.^15^ Cell-selective and temporally precise control over action potential generation in neural circuits, however, requires a specific knowledge and consideration of the tissue optical properties and illumination profiles.

Understanding and overcoming obstacles related to targeted illumination is therefore an essential step in designing effective *in vivo* experiments. A critical difference between the optogenetic stimulation in the brain and in the spinal cord lies in the light delivery system. In the brain, light can be delivered to any cortical or subcortical regions through a fiber-optic tip, often extended deep into tissue with minimal reported changes in animal behavior.^16^^–^^18^ This approach is unlikely to be successful in the spinal cord or periphery because an implanted optical fiber would severely damage the white matter tracts, which have minimal redundancy and carry high information density. Furthermore, the spinal cord is a much smaller and more mobile structure in comparison with the brain, making the targeting even more of a challenge. Light delivery systems are thus superficial, wrapping around a nerve, or fixed immediately dorsal to the spinal cord.^8^^,^^19^ As a consequence, the light source is placed relatively far from the target activation region and light must pass through the myelinated dorsal white matter of the spinal cord before reaching the opsin-expressing gray matter. Evaluating light propagation patterns is therefore critical for efficient optogenetic modulation.

While anisotropic light propagation has been observed in many biological tissues, an important piece of information that is currently missing is the directional dependence of light scatter in the highly organized spinal cord.^20^^,^^21^ Indeed, in the brain, it has been shown that nerve fiber orientation can induce a dramatic difference in effective attenuation depending on the angle of incident light with regards to the white matter tracts.^22^ Given this, and due to the fact that the spinal cord contains very highly organized white matter tracts extending the length of the vertebrae, a more in depth characterization of this directional effect was required.

To this end, we characterized the reduced scattering coefficient ($\mu_{s}^{\prime }$) in both the radial and longitudinal directions of the myelinated fibers in perfused macaque spinal cord, as well fixed and fresh human spinal cord. To achieve this, we use integrating sphere measurements and validate with microscope images and the “punch-through” method.^23^ Finally, we incorporate these data into a modified open-source three-dimensional (3-D) Monte Carlo program (mcxyz), allowing the use of multidimensional scattering coefficients, to visualize the anisotropic illumination profile in a typical optogenetic experiment.^24^

## 2 Results

We show herein that light propagation patterns in the spinal cord depend both on the local tissue scattering properties as well as the regional tissue organization.

### 2.1 Scattering Coefficient Depends on White Matter Tract Orientation

Spinal cord gray matter consists mainly of neuron and glial cell bodies along with nonuniform combinations of myelinated and nonmyelinated nerve fibers. On the other hand, spinal cord white matter is built up almost entirely of highly organized myelinated nerve fibers. While the anisotropic nature of neural tissue is often ignored, it is generally accepted that overall, the myelinated axons in white matter lead to increased scattering in comparison with gray matter.^25^^–^^29^ We show here that this is not always true and that the direction of light propagation with respect to white matter fiber tracts plays a profound role in the observed scattering and attenuation.

Specifically interesting, the white matter in the spinal cord has lower scattering in the longitudinal direction (along axons) and higher scattering in the radial direction (perpendicular to axons) compared with the gray matter present on the same slices. The results of these integrating sphere measurements are tabulated in Table 1. The difference in scattering power between the longitudinal and radial white matter was profound, with a 50% decrease measured in the longitudinal direction in all the tissues considered. The measurements in gray matter, however, produced similar results in both directions, suggesting that this effect arises due to the organized myelinated structures in white matter. While we measured $\mu_{s}^{\prime }$ in each Cartesian direction (x, y, and z), we reduce the terms to radial and longitudinal due to the symmetry of the organized myelin structures resulting in equal $\mu_{s_{x}}^{\prime }$ and $\mu_{s_{z}}^{\prime }$. Moreover, our results in radial white matter are very much in agreement with previous measurements of $\mu_{s}^{\prime }$ using a fiber optic probe.^30^ In all of our integrating sphere measurements, the measured absorption coefficient in all tissue regions and sections were below $0.1\;{\mathrm{mm}}^{-1}$. These measurements are purposefully taken at 633 nm to minimize absorption contributions, to better isolate and analyze directional scattering. While the main result here is the observed ratio of the scattering power between the two directions, the absolute values may also be useful due to limited reports in spinal cord tissue.

**Table 1:** Measured values of $\mu_{s}^{\prime }$ in the longitudinal and radial directions at 633 nm.

Species	Tissue type	$\mu_{s_{\text{Long}}}^{\prime }$ (1/mm)	$\mu_{s_{\mathrm{Rad}}}^{\prime }$ (1/mm)
Human (fresh)	White matter	1.56±0.2	3.51±0.3
	Gray matter	2.61±0.2	2.69±0.3
Human (fixed)	White matter	1.59±0.1	3.46±0.3
	Gray matter	2.24±0.2	2.57±0.2
Macaque (fixed)	White matter	1.58±0.2	3.59±0.3
	Gray matter	2.67±0.2	2.90±0.2

**Fig. 1:**
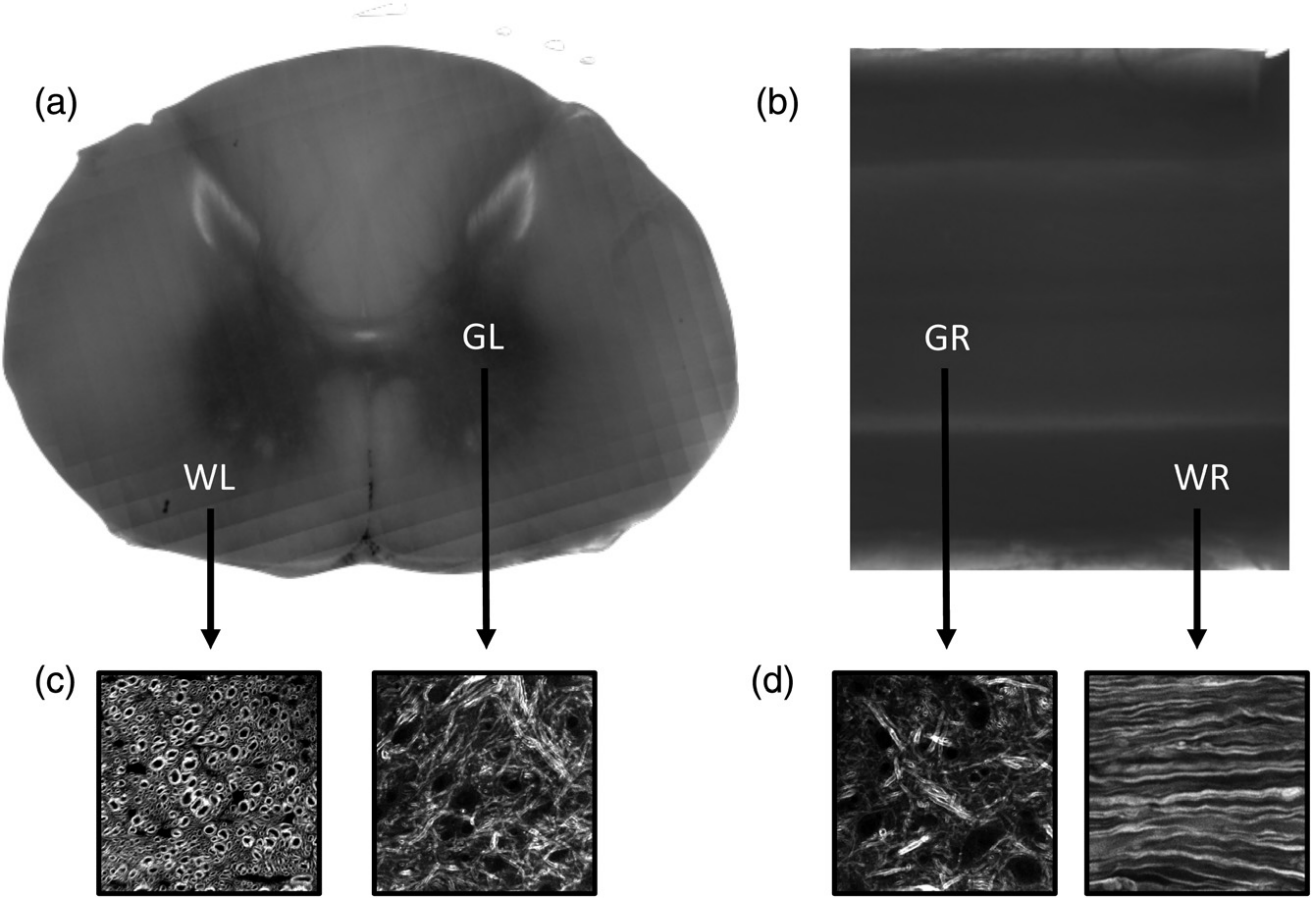
Transmission images of 1-mm spinal cord sections. (a) Transmission microscope image of 1-mm slice of macaque spinal cord in the longitudinal direction. There is higher transmission in white matter when imaging in this direction. (b) Transmission microscope image of 1 mm slice of macaque spinal cord in the radial direction. There is higher transmission in gray matter when imaging in this direction. (c) Backward-detected CARS images of white and gray matters in the longitudinal direction showing subcellular myelin composition. (d) Backward-detected CARS images of white and gray matters in the radial direction showing subcellular myelin composition. WL, white matter in longitudinal direction; GL, gray matter in longitudinal direction; WR, white matter in radial direction; GR, gray matter in radial direction.

We further validated the directional dependence of attenuation by imaging two 1-mm fixed macaque tissue samples in both the radial and longitudinal directions using a transmission microscope [Figs. 1(a) and 1(b)]. Indeed, we see the white matter regions have a directional dependence with respect to transmission, which is in good agreement with the inverse of the scattering relationship (white matter transmits more than gray matter in the longitudinal direction and less than gray matter in the radial direction). We also include images we have taken using coherent anti-Stokes Raman scattering (CARS) in primate spinal cord, in the various directions, showing the organization of the spinal cord segments at subcellular scale for reference [Figs. 1(c) and 1(d)].

As an additional and final validation that the reduced scattering coefficient is anisotropic in the white matter of the spinal cord, we performed the punch-through^23^ method. We experimentally estimate the relative values of $\mu_{\mathrm{eff}}$ in perfused mouse and macaque spinal cord white and gray matter sections, also in the longitudinal and radial directions. Due to the limited thickness of mouse spinal cord, it is not possible to obtain tissue samples for which the diffusion approximation is valid and $\mu_{\mathrm{eff}}=\sqrt{\mu_{a}(\mu_{a}+\mu_{s}(1-g)}$.^31^ Nevertheless, the initial exponential decrease is related to $\mu_{\mathrm{eff}}$. As seen in Table 2, we found the light traveling along the white matter tract axis is also roughly 50% less attenuated than light traveling perpendicular to the white matter tract. We again observed the lack of a directional preference in the gray matter structures, due once again to the lack of organized myelin. What is important to take away from these observations is the ratio of the coefficients between the two directions.

**Table 2:** Measured values for $\mu_{\mathrm{eff}}$ in the longitudinal and radial directions at 594 nm.

Species	Tissue type	$\mu_{{\mathrm{eff}}_{\text{Long}}}$ (1/mm)	$\mu_{{\mathrm{eff}}_{\mathrm{Rad}}}$ (1/mm)
Macaque (fixed)	White matter	1.90±0.2	3.83±0.45
	Gray matter	2.65±0.6	3.02±0.32
Mouse (fixed)	White matter	1.87±0.22	3.48±0.46
	Gray matter	2.46±0.16	2.36±0.32

## 3 Discussion

### 3.1 Simulating 3-D Optogenetic Activation Volumes Using Multidirectional Scattering Coefficients

**Fig. 2:**
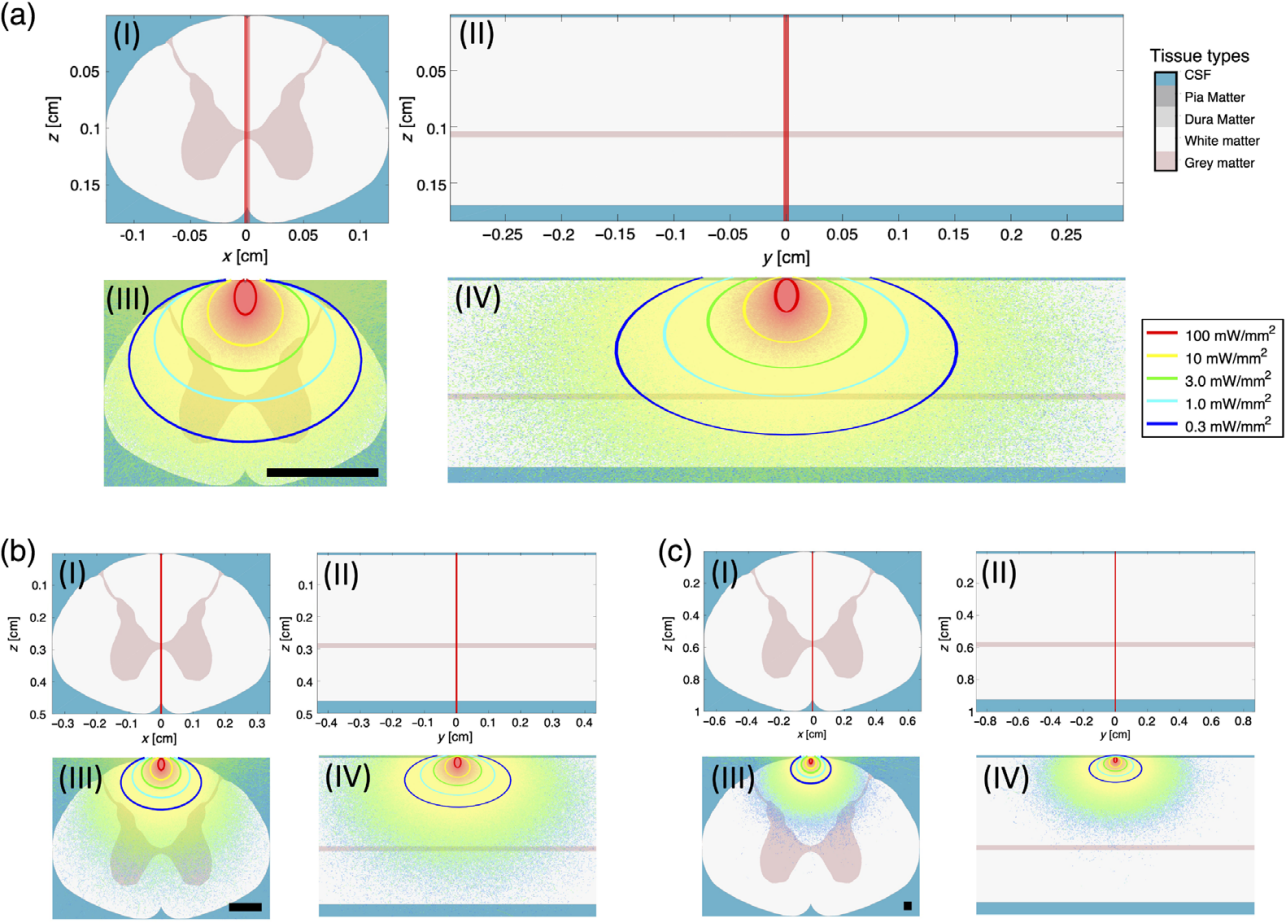
Monte Carlo simulation of fluence rate in different mammalian spinal cords using modified mcxyz. (a) Light deposition in mouse spinal cord. Also included here is the color-coded tissue type for each of the simulations and the legend for the optogenetic activation volume. (b) Light deposition in macaque spinal cord. (c) Light deposition in human spinal cord. (I) Tissue-type defined volume with photon trajectory in longitudinal plane. (II) Tissue-type defined volume with photon trajectory in radial plane. (III) Results of simulation in longitudinal plane with activation circles for optogenetic planning. (IV) Results of simulation in radial plane with activation circles for optogenetic planning. Input power is 10 mW in all simulations. Scale bars in (III) are 1 mm.

Using the different experimental evidences, we have shown that the scattering coefficient along the myelin fibers is approximately half the value obtained across the myelin fibers. Here, we investigate the impact on the light distribution in tissue by performing simplified light propagation simulations in spinal cord tissue volumes, showing the repercussions of directional light scattering on the optogenetic activation volume using an adjacent optical fiber illumination source. To do this, we have edited an existing open-source 3-D Monte Carlo package (mcxyz)^24^ to use multidirectional scattering coefficients (see Sec. 5 for more information). It is understood that it would require a complete treatment modeling the cylindrical structures of the white matter to obtain accurate simulations for these anisotropic structures. However, the focus is to obtain an estimate of the volume of tissue illuminated and the impact of an anisotropic scattering coefficient on that volume’s eccentricity. The results of these simulations with 10-mW laser power at 473 nm (channel rhodopsin excitation wavelength), in the various species’ spinal cords can be seen in Fig. 2. Described further in Sec. 5, one of the reasons for using the mcxyz program as a base for our modification is the package’s ability to generate generic tissue volumes with user-defined chromophore absorption (i.e., hemoglobin), at any given wavelength, as well as its ability to automatically calculate the relative scattering coefficient at any wavelength (herein 473 nm) in reference to a measured one (herein 633 nm).

The spinal cords are to scale in this simulation and we can see here the obvious difficulties and importance of accurate planning in translating optogenetics to larger animals. For the case of the mouse simulation, $\mu_{s}^{\prime }$ values from the macaque spinal cord were used, as small size made it too difficult to separately measure $\mu_{s}^{\prime }$ in the white and gray matter regions. We believe this to be a satisfactory assumption based on the similarity in measured $\mu_{\mathrm{eff}}$ in the macaque and mouse spinal cord. To elaborate on the implications of illumination volume in the mouse spinal cord, 10 mW is enough optical power to activate channel rhodopsin (activation intensity: $1\;{\mathrm{mW}/\mathrm{mm}}^{2}$)^32^^,^^33^ in every laminae down to the upper edges of the laminae 7. This corresponds to about 1.2 mm of tissue being activated in the dorsal to ventral direction, when the fiber output is perpendicular to the myelin sheaths. In the longitudinal direction, with the same fiber orientation, channel rhodopsin can be activated up to 1.6 mm away from the fiber tip’s midline, in both directions. A cervical vertebrae length in mice is about 800  μm, meaning that effective simulations that take into account anisotropic scattering are important for optogenetic planning, especially if only specific vertebrae are to be optogenetically investigated.^34^ In the macaque and human, this illumination scheme does not penetrate deep enough to activate even the first laminae. While increasing input power is an option, more elaborate fiber optic placement will become a critical factor in planning the optogenetic experiments in larger animals, and the observations provided herein will be useful for this.

### 3.2 Myelinated Axons May Act as Naturally Occurring Optical Waveguides

Perhaps the most interesting theory for the observed anisotropy was proposed by Kumar et al., who put forth the idea of myelin sheaths acting as natural optical waveguides.^35^ While their hypothesis was only investigated theoretically, we believe this to be a plausible answer. As an initial test, we imaged a 500-μm and 1-mm-thick longitudinal spinal cord section in a transmission arrangement to see if the myelin sheaths were indeed visible and more bright than the axon core. At this thickness, without any waveguiding effects the sheaths should not be visible due to multiple scattering events. However, we did indeed observe myelin rings appearing brighter than the axon cores in the transmission orientation (see Appendix 1 for more information). These results, while not conclusive, suggest a clear need for further investigation of the waveguide hypothesis. Similarly, if a waveguiding effect is confirmed, it would indicate that the scattering coefficients we measured are not true reduced scattering coefficients since guiding effects are not assumed to be present for their derivations.

### 3.3 Tissue Fixation Caused Negligible Effects in Data Analysis

In our work, we used perfused, paraformaldehyde (PFA)-fixed tissue to explicitly remove any hemoglobin absorption that could be present in the measurements. This allowed us to directly compare the attenuation and scattering power of the tissue in the different directions without compounding factors. While tissue fixation has been reported to change the optical properties of the sample, at 633 nm, the effects of formalin fixation have been shown to be minimal both in terms of birefringence profiles and measured scattering power.^36^^,^^37^ We strengthen this claim by comparing our fresh and fixed human tissue measurements using the integrating sphere setup (see Table 1 for results), showing that fixation does not significantly alter the absolute values or directional ratio of the measured reduced scattering coefficients at this wavelength.

### 3.4 Spinal Cord Measurements Along with DTI Tractography of the Brain Could Make Present Myelin Information Translatable

Due to the symmetry of the myelin sheath and the spinal cord organization, we use the terminology radial and longitudinal rather than Cartesian units for $\mu_{s}^{\prime }$ in x, y, z. We believe that this representation is more translational for expanding the technique to the brain along with diffuse tensor imaging (DTI) of white matter tractography.^38^

Some work has been done in measuring the optical properties of brain tissue in all 3-D in rat using discrete raster-scanned slices with a similar integrating sphere arrangement used here.^39^ This could offer an interesting opportunity to compare a full 3-D Monte Carlo simulation using these measured values for $\mu_{s}^{\prime }$ in x, y, z with a two-dimensional (2-D) symmetry-inspired $\mu_{s}^{\prime }$ simulation in just the radial and longitudinal directions using noninvasive DTI, in the future goal of patient-specific simulations. This ability could improve both optogenetic experiments and clinical technologies, such as tissue oximeters.

## 4 Conclusion

We present here experimental evidence of a directional dependence of light propagation in the white matter of the spinal cord. We also show that this effect is not species dependent and that the reduced scattering coefficient is ∼50% smaller along the myelin fibers compared with perpendicular to these fibers. This results in a light distribution that extends 20% further along the fibers, when the input beam is perpendicular to them. Using the information we present, we believe that improved Monte Carlo simulations for both stimulating and sensing endeavors can be achieved. This will be especially important for optogenetic planning wherein the experiment requires a specific laminae to be reached, or where the light must remain local to a specific spinal region (i.e., cervical region). While the work we present here is in the spinal cord, due to the symmetry of myelin, the information can also be used to improve optical simulations in the brain, using information from routine DTI tractography.

## 5 Methods

### 5.1 Scattering and Absorption Coefficient Calculation

The scattering and absorption coefficients were acquired using double-integrating-sphere setups, which measure the total diffuse reflection and transmission of a laser beam traveling through the tissue sample located between the two spheres. These measurements were performed on fixed macaque and human spinal cord tissue as well as fresh human tissue. This technique is routine and is explained in detail elsewhere.^40^ All tissue samples were cut in 1-mm intervals in both the longitudinal direction (along length of the spinal cord) and in the radial direction (along width of spinal cord) and measured along the two directions using the integrating sphere arrangement. With the measures of the integrating spheres, the inverse adding doubling (IAD) algorithm was performed to estimate the optical properties of the tissue under investigation. Also a routine technique, the procedure and theory of the calculation can be read elsewhere.^41^ Attention was given to assure the tissue sections were indeed homogeneous in the areas of measurements, as is a requirement for IAD. The laser wavelength used for these measurements was 633 nm and the beam spot was about 1 mm. The laser wavelength was specifically chosen to diminish absorption effects caused by hemoglobin as to target the directional dependence of scattering.

### 5.2 Attenuation Coefficient Estimation

**Fig. 3:**
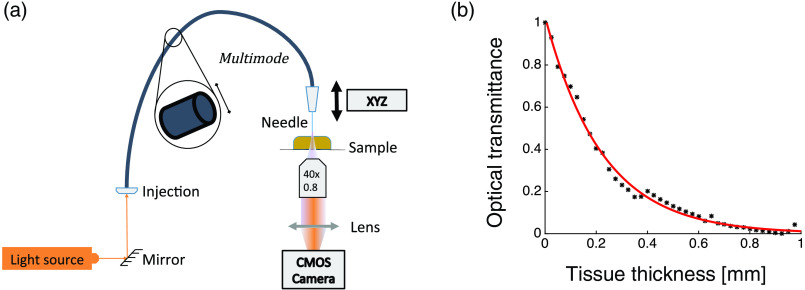
Punch-through method. (a) Spinal cord slices were illuminated by 594-nm laser beam coupled to a multimode optical fiber. The fiber was lowered into the tissue and the light transmitted through a slide was collected on the underside by a low numerical aperture objective and detected using a CMOS camera. (b) Optical transmittance as a function of tissue thickness. Experimental measurements (black) and exponential fit (red line).

Light transmission measurements were conducted with mouse and macaque spinal cord slices using a fiber punch-through method.^23^ 594-nm (MBL-FN, Changchun New Industries Optoelectronics Technology, China) laser light was delivered to a 105-μm-diameter optical fiber (FG105UCA, Thorlabs). The fiber tip was then lowered into the tissue in 5  μm steps, and the light intensity was collected on the underside of the slice by a low numerical aperture (N.A.) objective (10×, 0.25NA) and recorded by a CMOS camera (DMK23UP031, Imaging Source), seen in Fig. 3(a). Optical transmittance, the amount of light transmitted through a slice, decays exponentially with the tissue thickness and thereby its evolution can be parameterized by fitting the observed intensity profiles with a single exponential function, as seen in Fig. 3(b).

#### 5.2.1 Data analysis

Optical transmittance, the amount of light transmitted through a slice, was calculated as a ratio of the total integrated intensity (summed over all pixels in the image) at a given depth over the optical fiber intensity with no tissue present. Transmission of light through the tissue was then plotted as a function of the tissue thickness and parameterized by fitting the observed intensity profiles with a single exponential function. In the diffusion regime, the light can be modeled by the modified Beer–Lambert law^23^

$$\frac{I(z)}{I_{0}}=T(z)=\exp(-\mu_{\mathrm{eff}}z),$$ (1)

with I(z) being the total intensity detected by camera, z being the tissue thickness, and $I_{0}$ being the intensity at the fiber tip. T(z) is the optical transmittance, which follows an exponential decay against the tissue thickness. In our experiment, we do not reach the diffusion regime in the case of the mouse tissue because the spinal cord sections are simply too small (therefore, $\mu_{\mathrm{eff}}\neq \sqrt{3\mu_{a}[\mu_{a}+\mu_{s}(1-g)]}$). Nevertheless, the exponential coefficient is obtained as a relative value of $\mu_{\mathrm{eff}}$ as it is related to the attenuation.

### 5.3 3-D Monte Carlo Simulations with Multidirectional Scattering Coefficients

We achieve the simulations using an altered 3-D Monte Carlo program (mcxyz) provided open-source by Jacques, used previously in the brain.^24^^,^^39^ This program is a verified variation of the 2-D Monte Carlo program created for multilayered tissue (MCML), used routinely for biological applications.^42^ Using a simple segmentation program, we converted our spinal cord models from an image stack to a tissue-type-defined 3-D volume for the simulation.

An immense advantage of the mcxyz program is the inclusion of Jacques’s method for creating generic tissues, allowing a highly malleable simulation based on few input parameters.^25^ First and foremost, it allows the ability to use a measured scattering coefficient acquired at any visible or NIR wavelength (in our case 633 nm) and to then perform a simulation at another wavelength by calculating a scattering coefficient relative to that point (i.e., 473 nm). Similarly, while the measurements are also made at a lower absorbing wavelength and often in bloodless tissue, mcxyz allows us to simply add an estimated blood concentration to each tissue type separately, for the inclusion of hemoglobin absorption at the simulation wavelength. This has previously been used for simulations in the brain.^43^ Ultimately, this allows us to simulate the light propagation in living tissue at traditional optogenetic activation wavelengths. Lastly, the program allows to input the light source properties such as: angle of incidence, incident laser wavelength, beam divergence, and beam starting position. We display herein the case of a 105-μm multimode optical fiber with a N.A. of 0.22 situated against and perpendicular to the white matter tracts in the center of the dorsal spinal cord (Fig. 2). The laser wavelength used in this simulation was 473 nm (to make the simulation relevant to those using channel rhodopsin) and a blood volume of 2.8% with an oxygen saturation: 62% was deployed, as was described for brain tissue in other work.^25^^,^^43^

To accommodate our findings of a directional scattering dependence, we included a small alteration in the Monte Carlo action of the mcxyz program where rather than having a sole scattering coefficient for each pixel in the volume, there exists one for each 3-D direction. During the process of the photon step, where the scattering coefficient is considered, the three pixel-corresponding scattering coefficients are linearly combined using the unit vector of the photon’s current propagating direction, and from there the program resumes normally. The modified version has been validated to give identical results to the original mcxyz code when all 3-D scattering coefficients are equal.

### 5.4 Sample Preparation

All animal tissues were obtained according to protocols that had been approved by the Institutional Animal Care and Use Committee (Comité de Protection des Animaux de l’Université Laval), and all procedures involving animals and their care were made in accordance with the Canadian Council on Animal Care’s Guide to the Care and Use of Experimental Animals. Human postmortem brain tissues were obtained from the human brain bank of the Centre de Recherche de l’Institut Universitaire en Santé Mentale de Québec (CERVO), which required informed consent before donation of tissues. CERVO’s Ethics Committee approved the brain collection, storage, and handling procedures, which were described in detail elsewhere.^44^

#### 5.4.1 Mice and monkeys

Three mice and three macaque specimens were anesthetized with urethane (2  g/kg) and perfused intracardially with 4% PFA in 0.1 M phosphate buffer; spinal cords were postfixed overnight in the same solution at 4°C. The tissue was then stored in a 0.1 M phosphate buffer solution. The slices were embedded in agarose and cut in parasagittal and transversal planes at varying thicknesses. Before measurements, the tissue was always thoroughly rinsed in a water bath for 2 h.

#### 5.4.2 Human

Two cervical sections of the same human spinal cord were extracted upon the arrival of newly postmortem tissue at the brain bank. The first section was fixed in a 4% PFA bath, whereas the other was immediately used for optical analysis. To slice the sections, they were embedded in agarose and cut in parasagittal and transversal planes at 1 mm thicknesses.

## 6 Appendix 1

### 6.1 Myelin May Act as an Optical Waveguide

**Fig. 4:**
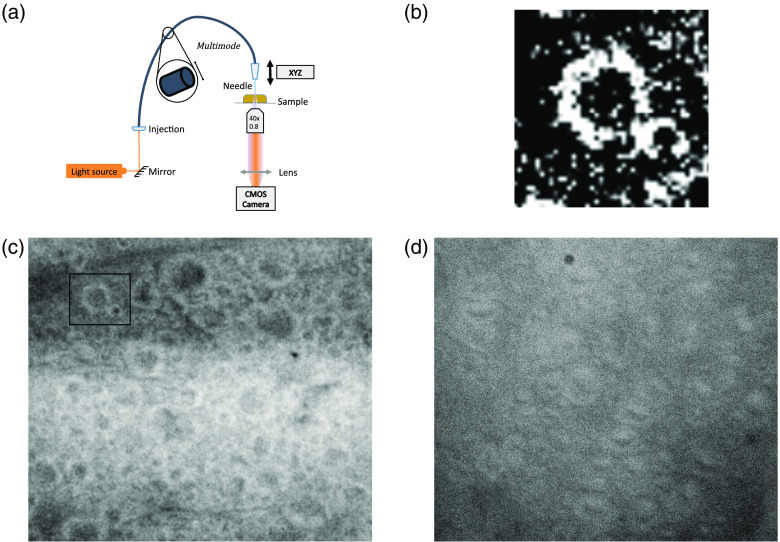
Possible transmission of light through myelin sheaths in thick longitudinal slices of spinal cord. (a) Optical arrangement for the imaging experiment. Light was collected on the underside of the slice by a 40× objective and recorded by a CMOS cameras (DMK 23UP031, imaging source). (b) Magnified and contrasted image of bright myelin sheath and dark axon center from (c). (c) 500-μm longitudinal slice illuminated using a 594-nm fiber laser source (MBL-FN, Changchun New Industries Optoelectronics Technology, China). Inset square shows location of zoomed image in (b). (d) 1-mm longitudinal slice illuminated with a fibered white lamp source (SLS201L, Thorlabs, New Jersey).

Recently, it has been theorized that myelin sheaths may act as natural optical waveguides.^35^ To experimentally investigate this we imaged thick sections of longitudinal spinal cord where the myelin sheaths are highly organized at high magnification, in a transmission arrangement. While we have not exhausted the possibilities of other optical effects, it looks as though the myelin is indeed transmitting more light in the sheath, as opposed to the axon, which matches the theoretical investigation (see Fig. 4). We tried two different thicknesses and two different sources, and the pattern of brighter myelin sheaths remained in all cases.
